# Treatment of hepatitis C virus genotype 3 infection with direct-acting antiviral agents

**DOI:** 10.1590/1414-431X20165504

**Published:** 2016-10-24

**Authors:** L.P. Zanaga, N. Miotto, L.C. Mendes, R.S.B. Stucchi, A.G. Vigani

**Affiliations:** Divisão de Moléstias Infecciosas Departamento de Clínica Médica, Universidade Estadual de Campinas, Campinas, SP, Brasil

**Keywords:** Hepatitis C treatment, Genotype 3, Sofosbuvir, Daclatasvir, Ribavirin

## Abstract

Hepatitis C virus (HCV) genotype 3 is responsible for 30.1% of chronic hepatitis C infection cases worldwide. In the era of direct-acting antivirals, these patients have become one of the most challenging to treat, due to fewer effective drug options, higher risk of developing cirrhosis and hepatocellular carcinoma and lower sustained virological response (SVR) rates. Currently there are 4 recommended drugs for the treatment of HCV genotype 3: pegylated interferon (PegIFN), sofosbuvir (SOF), daclatasvir (DCV) and ribavirin (RBV). Treatment with PegIFN, SOF and RBV for 12 weeks has an overall SVR rate of 83–100%, without significant differences among cirrhotic and non-cirrhotic patients. However, this therapeutic regimen has several contraindications and can cause significant adverse events, which can reduce adherence and impair SVR rates. SOF plus RBV for 24 weeks is another treatment option, with SVR rates of 82–96% among patients without cirrhosis and 62–92% among those with cirrhosis. Finally, SOF plus DCV provides 94–97% SVR rates in non-cirrhotic patients, but 59–69% in those with cirrhosis. The addition of RBV to the regimen of SOF plus DCV increases the SVR rates in cirrhotic patients above 80%, and extending treatment to 24 weeks raises SVR to 90%. The ideal duration of therapy is still under investigation. For cirrhotic patients, the optimal duration, or even the best regimen, is still uncertain. Further studies are necessary to clarify the best regimen to treat HCV genotype 3 infection.

## Introduction

The hepatitis C virus (HCV) comprises six genotypes and multiple subtypes (1). HCV genotype 1 is the most prevalent worldwide, accounting for 83.4 million infections (46.2% of all HCV patients) and is the most prevalent in the Americas, Europe and Australia (2). Genotype 3 is the second most prevalent globally (54.3 million patients, 30.1%); genotypes 2, 4, and 6 are responsible for 22.8% and genotype 5 comprises the remaining less than 1% of patients (2).

Evidence that HCV genotype 3 increases the risk of progression to cirrhosis or to hepatocellular carcinoma (HCC) was published in the last years. The Swiss Hepatitis C Cohort Study found that the most significant independent risk factors associated with accelerated liver fibrosis progression, in a multivariate model analysis, were histological activity [OR=2.03 (1.54–2.68), P<0.001], genotype 3 infection [OR=1.89 (1.37–2.61), P<0.001], male sex [OR=1.60 (95%CI=1.21–2.12), P<0.001] and age at infection [OR=1.08 (1.06–1.09), P<0.001] (3). Among patients with HCV infection and cirrhosis, genotype 3 infection is also the strongest predictor for the occurrence of HCC. In a French cohort, the rate of HCC occurrence after 5 years was 34% among those with cirrhosis due to chronic HCV genotype 3, and 17% in other genotypes (P=0.013) (4).

Until 2011, the only treatment options available for patients with HCV infection were interferon-based regimens, with pegylated interferon alfa 2a or 2b (PegIFN) and ribavirin (RBV). In 2011 the first direct-acting antiviral agents (DAAs), NS3/4A protease inhibitors (telaprevir and boceprevir) became available for HCV genotype 1 infection and their association with PegIFN/RBV improved the sustained virological response (SVR) rates (5,6). However, NS3/4A protease inhibitors have limited activity against HCV genotype 3, even considering second-generation drugs such as simeprevir and paritaprevir.

In 2013, new DAAs became available for HCV infection treatment, though few are effective for HCV genotype 3 infection, such as daclatasvir (DCV) and sofosbuvir (SOF). SOF is a pyrimidine nucleotide analogue inhibitor of the HCV RNA-dependent RNA polymerase, with excellent antiviral activity against all HCV genotypes and a high genetic barrier to resistance (7). DCV is a HCV NS5A inhibitor that reduces the assembly of the replication complex and also has antiviral activity across all genotypes (8). NS5A is a zinc-binding phosphoprotein that plays an important, although not totally clear, role in HCV replication.

Currently, only four drugs (PegIFN, RBV, SOF and DCV) are recommended by the European Association for the Study of the Liver (EASL) and the American Association for the Study of Liver Diseases (AASLD) for the treatment of HCV genotype 3 infection (9,10). As a consequence, a small number of effective treatment options are available for patients infected by HCV genotype 3. The EASL guidelines recommend six treatment options for patients infected with HCV genotype 1, and only three for genotype 3 (9). Similarly, AASLD recommends four treatment options for patients infected with HCV genotype 1, while for genotype 3 there are only two therapeutic regimens recommended and one alternative option (10).

In addition, the first interferon-free regimen available for genotype 3 treatment, SOF plus RBV for 24 weeks, showed a remarkably low SVR rate of 62% for cirrhotic patients who have not responded previously to PegIFN/RBV therapy (11).

At the moment, patients with HCV genotype 3 infections are considered a special population and have become one of the most challenging subpopulations to treat. Studies have shown that genotype 3 is associated with faster progression to cirrhosis and, thus, has a higher likelihood of hepatocellular carcinoma in comparison to the other genotypes (3,4). In addition, few effective treatment options are available and the use of some therapeutic options is not yet supported by clinical trial data in this subset of patients. Finally, patients with HCV genotype 3 infection and cirrhosis, especially those who are treatment-experienced, have the lowest SVRs in the DAA era.

Given this context, this article reviews the combinations of drugs that can be used for the treatment of HCV genotype 3 infection with and without cirrhosis.

## Material and Methods

A PubMed search was undertaken to identify relevant literature using search terms including “hepatitis C treatment”, “HCV treatment”, “HCV genotype 3”, “HCV genotype 3 therapy”, “sofosbuvir” and “daclatasvir”. Furthermore, abstracts presented at recent international meetings in liver disease, viral hepatitis and infectious disease, as well as the reference lists of the AASLD and EASL guidelines were searched to identify publications not retrieved by PubMed searches.

## Treatment of HCV genotype 3 infection in non-cirrhotic patients: sofosbuvir plus ribavirin

The combination of SOF plus RBV for 24 weeks was the first interferon-free therapy for patients with HCV genotype 3 infection approved by the FDA. International guidelines differ regarding the recommendations for this regimen. EASL guidelines do not recommend this therapeutic regimen for treatment-experienced cirrhotic patients. On the other hand, AASLD recommends SOF plus RBV as an alternative regimen for patients without cirrhosis with previous PegIFN/RBV failure or treatment-naive patients who are IFN-ineligible (9,10).

In naive non-cirrhotic patients with HCV genotype 3 infection, SOF plus RBV for 12 weeks resulted in an overall SVR of 61-68%. However, extending the treatment to 24 weeks led to an approximate 30% increase in SVR rates, ranging from 90 to 96% (Table 1).

**Table:**
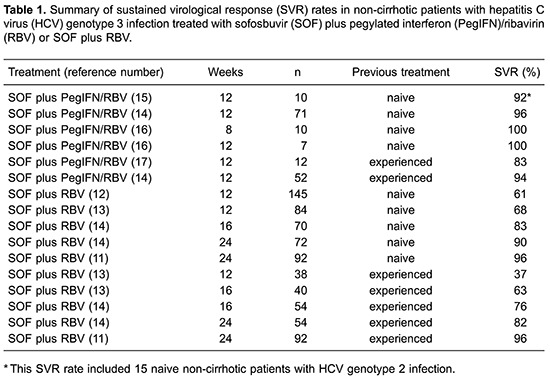

Two large clinical trials evaluated the efficacy of SOF plus RBV for 12 weeks in naive, non-cirrhotic patients infected with HCV genotype 3. The Fission trial included 145 patients, but only 89 (61%) achieved SVR (12). The Positron trial included 84 naive patients who were interferon-ineligible or intolerant, of which 57 (68%) reached SVR (13). The Boson clinical trial found higher SVR among naive and non-cirrhotic patients treated with SOF plus RBV for 16 weeks. Among 70 patients treated, 58 (83%) achieved SVR (14). Another arm of this study evaluated 72 naive non-cirrhotic patients treated for 24 weeks, with an overall SVR of 90% (65 patients). SOF plus RBV for 24 weeks was also used in the Valence trial, which included 92 patients and 87 (96%) achieved SVR (11).

In treatment-experienced, non-cirrhotic patients with HCV genotype 3, SOF plus RBV for 12 weeks resulted in low SVR rates (37%), similar to what was observed in the naive population. Extending treatment to 24 weeks led to an approximate 50% increase in SVR rates, ranging from 82 to 96% (Table 1). The Fusion trial investigated 12 or 16 weeks of SOF plus RBV in previous treatment-experienced non-cirrhotic patients with HCV genotype 3 infection (13). Thirty eight patients were treated for 12 weeks and 40 patients for 16 weeks and the SVR rates were, respectively, 38% (14 of 38) and 63% (25 of 40). The Boson study also evaluated SOF plus RBV for 16 weeks in 54 non-cirrhotic patients and previous non-responders to PegIFN/RBV, with 41 (76%) achieving SVR (14).

Finally, two studies evaluated SOF plus RBV for 24 weeks in non-cirrhotic patients and previous non-responders to PegIFN/RBV. The Boson study included 54 patients, 44 (82%) of whom achieved SVR (14). The Valence study included 92 patients and 87 (96%) achieved SVR (11).

In conclusion, combination therapy of SOF plus RBV for 24 weeks has been shown to yield a high SVR rate in naive patients or previous PegIFN/RBV non-responders with HCV genotype 3 without cirrhosis. Shorter therapies have not been able to reach acceptable SVR rates.

## Sofosbuvir plus pegylated interferon/ribavirin

The combination of SOF plus PegIFN/RBV for 12 weeks is the only interferon based therapy recommended by the EASL and AASLD guidelines for the treatment of HCV genotype 3 infection (9,10).

In naive non-cirrhotic patients, SOF plus PegIFN/RBV for 12 weeks resulted in an overall SVR of 92-100% (Table 1). However, efficacy data is scarce: few patients were included in clinical trials and only three studies (two phase II studies and one phase III study) evaluated the SVR rates in this population. The phase II study included 25 naive non-cirrhotic patients (15 with HCV genotype 2 and 10 with genotype 3 infection) treated with SOF plus PegIFN/RBV for 12 weeks, reaching an overall SVR rate of 92%, but no SVR data according to specific genotype is available (15). Another phase II study included 17 patients treated with SOF plus PegIFN/RBV for either 12 (7 patients) or 8 weeks (10 patients) and the overall SVR rate was 100% in both arms (16). The Boson phase III study included 71 naive non-cirrhotic patients with HCV genotype 3 infection treated with SOF plus PegIFN/RBV for 12 weeks, achieving an overall SVR rate of 96% (14).

In treatment-experienced non-cirrhotic patients, SOF plus PegIFN/RBV for 12 weeks resulted in overall SVR rates of 83-94% (Table 1), although these results are supported by data from only two studies with 64 patients. Lonestar, a phase II study, included only 12 patients without cirrhosis, of which 10 (83%) achieved SVR, one relapsed and the other was lost to follow-up (17). Boson, a phase III study, included 52 non-cirrhotic patients with genotype 3 HCV infection and previous failure to PegIFN/RBV, with an overall SVR rate of 94% (14).

In non-cirrhotic patients, including naive and with previous failure to PegIFN/RBV, SOF plus PegIFN/RBV for 12 weeks resulted in high SVR rates (83–100%) (Table 1). It must be noted that non-significant differences in SVR rates were observed among naive and treatment-experienced patients, but these data need to be cautiously analyzed, since only small cohorts were included in the studies.

## Sofosbuvir plus daclatasvir with or without RBV

The combination SOF plus DCV for 12 weeks is recommended by EASL and AASLD for the treatment of patients with HCV genotype 3 infection (9,10). In non-cirrhotic patients with HCV genotype 3 infection, whether naive or treatment-experienced, SOF plus DCV with or without RBV for 12 or 24 weeks resulted in an overall SVR of 80–100% (Table 2).

**Table:**
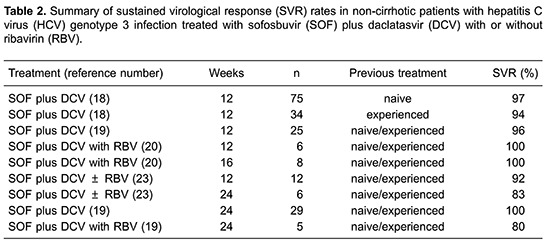

Among naive or treatment-experienced patients without cirrhosis, treatment with SOF plus DCV for 12 weeks resulted in an overall SVR of 94–97% (Table 2). Two studies evaluated SOF plus DCV for 12 weeks in naive or treatment-experienced non-cirrhotic patients with HCV genotype 3 infection (18,19). ALLY-3, a phase III clinical trial, included 75 naive and 34 previously treatment-experienced patients and SVR rates were, respectively, 97% (73 of 75) and 94% (22 of 34) (Table 2) (19). An observational study included 25 naive and treatment-experienced patients, 24 of whom (96%) achieved SVR (Table 2) (19).

A single study, ALLY-3+, evaluated the addition of RBV to SOF plus DCV for 12 or 16 weeks for HCV genotype 3 naive or treatment-experienced patients without cirrhosis, including 14 patients with advanced fibrosis, but without cirrhosis. Six patients were treated for 12 weeks and 8 patients were treated for 16 weeks with SOF plus DCV and RBV, with all of them achieving SVR12 in both the 12- and 16-week treatment arms (20).

Another study evaluated SOF plus DCV with or without RBV for 12 or 24 weeks in naive or treatment-experienced patients with HCV genotype 3 (21). The SVR rates were, respectively, 92% (11 of 12) with 12 weeks of treatment and 83% (5 of 6) with longer treatment for 24 weeks (21). Therefore, the addition of RBV did not seem to improve the outcome of therapy, since high SVR rates were observed in patients treated with SOF plus DCV with or without RBV, but both treatment arms included only a small number of patients.

Finally, one observational study assessed the SVR rates in patients treated for 24 weeks with SOF plus DCV with or without RBV (19). Among the 29 patients treated with SOF plus DCV without RBV, 29 (100%) achieved SVR, while among 5 patients treated with SOF plus DCV with the addition of RBV for 24 weeks, 4 (80%) achieved SVR.

To summarize, the addition of RBV for non-cirrhotic genotype 3 patients treated with SOF plus DCV did not alter SVR rates significantly. Increasing the duration of treatment from 12 to 24 weeks also did not result in higher SVR rates. It is important to emphasize that the SVR rates discussed above are based on few clinical trials and observational studies that included small populations.

## Treatment of HCV genotype 3 with compensated cirrhosis: sofosbuvir plus ribavirin

SOF plus RBV for 12 weeks is not recommended for treatment of cirrhotic patients with HCV genotype 3 infections. The overall SVR rates in naive cirrhotic patients treated for 12 weeks ranged from 21 to 34% (Table 3). Two trials found that SOF plus RBV for 12 weeks for naive patients with cirrhosis resulted in SVR rates of 21% (3 of 14) and 34% (13 of 38) (12,13).

**Table:**
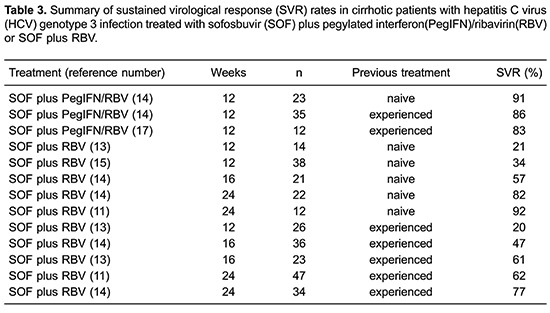

Extending the treatment with SOF plus RBV in this population improved the SVR rates (Table 3). The Boson study included 21 naive patients with cirrhosis treated with SOF plus RBV for 16 weeks and 12 (57%) achieved SVR (14). Extending the treatment for 24 weeks in this population improved the SVR rates from 82 to 92% (Table 3). In the Boson study, among 22 naive patients with cirrhosis treated with SOF plus RBV for 24 weeks, 18 (82%) achieved SVR (14). In the Valence study, 12 of 13 patients (92%) achieved SVR (11). SOF plus RBV for 24 weeks is recommended by EASL for treatment-naive patients with cirrhosis and by AASLD as an alternative regimen for treatment-naive patients with HCV genotype 3 infection who are IFN-ineligible (9,10).

Among treatment-experienced patients with cirrhosis and HCV genotype 3 infection treated with SOF plus RBV for 12 weeks, the SVR rates are around 20%, similar to those observed in naive patients (Table 3). Only one study, the Fusion trial, assessed SOF plus RBV for 12 weeks in treatment-experienced patients with cirrhosis, including 26 patients, and only 20% achieved SVR (13).

In this population, as observed in naive patients, extending the treatment duration with SOF plus RBV improved the SVR rates. Two studies evaluated SOF plus RBV for 16 weeks in treatment-experienced patients. The Boson study included 36 patients, reaching 47% SVR (17 patients) (14). The Fusion study included 23 patients, of which 14 (61%) achieved SVR (13). Finally, two other studies evaluated the extension of the treatment regimen with SOF plus RBV from 12 to 24 weeks in this population. In the first study only 60% (29 of 47) of prior treatment-experienced patients achieved SVR (11). The other study, Boson, comprising 34 patients, had a 77% SVR rate (20 patients) (14). SOF plus RBV for 24 weeks in treatment-experienced patients with cirrhosis resulted in low SVR rates, below 80%. Therefore, the EASL and the AASLD guidelines do not recommend this therapeutic regimen for treatment-experienced cirrhotic patients with HCV genotype 3 infection (9,10).

## Sofosbuvir plus and ribavirin

The combination SOF plus PegIFN/RBV for 12 weeks is recommended by EASL and AASLD for the treatment of naive or treatment-experienced patients with compensated cirrhosis and HCV genotype 3 infection (9,10). This recommendation is based in only one study, which observed an overall SVR rate of 86–92% in compensated cirrhotic patients (Table 3).

The Boson phase III study found SVR rates of 91% for the treatment of 23 naive patients with compensated cirrhosis with SOF plus PegIFN/RBV for 12 weeks (14). The treatment-experienced cirrhotic population was evaluated in two studies, which assessed the SVR rates with SOF plus PegIFN/RBV for 12 weeks (14,17). The Boson study included 35 patients and 30 (86%) achieved SVR (14). Lonestar, a phase II study, included 12 treatment-experienced cirrhotic patients and ten (92%) reached SVR (17).

Even though it was only evaluated in small cohorts, the regimen containing SOF plus PegIFN/RBV for 12 weeks presents an adequate option for patients with compensated cirrhosis; however, interferon-based therapy may have some contraindications and is also associated with high rates of adverse events, especially in the cirrhotic population, justifying the ongoing search of safer and more effective therapies for these patients.

## Sofosbuvir plus daclatasvir with or without RBV

The combination of SOF plus DCV for 24 weeks is recommended by the EASL and the AASLD guidelines for the treatment of cirrhotic patients with HCV genotype 3 infection (9,10). In naive or treatment-experienced patients with cirrhosis, 12 weeks of SOF plus DCV or SOF plus DCV and RBV resulted in overall SVR rates of 58–69% and 80–83%, respectively (Table 4). Furthermore, the extension of the treatment to 24 weeks with SOF plus DCV or SOF plus DCV and RBV resulted in increased overall SVR rates of 90–100% and 85%, respectively (Table 4).

**Table:**
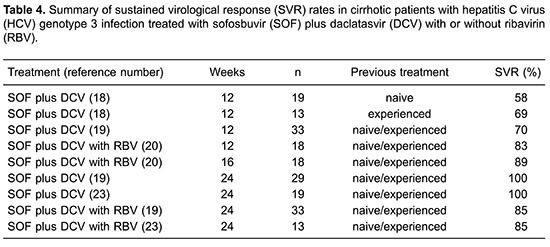

Two studies assessed the SVR rates in naive or treatment-experienced cirrhotic patients treated with SOF plus DCV for 12 weeks. ALLY-3 included 19 naive and 13 treatment-experienced patients with cirrhosis treated with SOF plus DCV for 12 weeks, with SVR rates of, respectively, 58% (11 of 19) and 69% (9 of 13) (18). An observational study included 30 patients with compensated cirrhosis, naive or previously treatment-experienced (77% had previously been treated with PegIFN plus RBV), who were treated with SOF plus DCV (only 4 patients received RBV) for 12 weeks and 24 (80%) achieved SVR (19).

ALLY-3+ evaluated SVR rates with SOF plus DCV and RBV for 12 weeks and the extension of the treatment to 16 weeks in naive or experienced patients. This study included 18 patients treated with SOF plus DCV with RBV for 12 weeks and 18 patients treated for 16 weeks, observing SVR rates of, respectively, 83% (15 of 18) and 89% (16 of 18) (20).

Two observational studies evaluated SVR rates among naive or experienced patients with cirrhosis treated with SOF plus DCV for 24 weeks (19,22). One study included 100 patients and 90 (90%) achieved SVR (19). Another study included only 19 patients and all achieved SVR (22). Other observational studies evaluated SOF plus DCV with the addition of RBV for 24 weeks. In one study, 33 patients were included and 28 (85%) achieved SVR while in the other study, among 13 patients treated with SOF/DCV for 24 weeks with the addition of RBV, 11 (85%) achieved SVR (19,23).

In conclusion, SOF plus DCV without RBV for 12 weeks had unacceptably low SVR rates, overall below 70% in cirrhotic patients with HCV genotype 3 infection. However, the addition of RBV improves SVR rates up to 80%. Furthermore, in this population the highest SVR rates, ranging from 85 to 100%, were observed with SOF plus DCV with RBV for 24 weeks, although stronger recommendations are not possible, since most studies were observational and included only a small number of patients.

## Treatment of HCV genotype 3 infection with decompensated cirrhosis with sofosbuvir and daclatasvir

Patients with HCV genotype 3 infection and decompensated cirrhosis have only one treatment recommendation according to the AASLD and EASL guidelines: SOF plus DCV for 12 or 24 weeks, with or without RBV. EASL recommended that patients with decompensated cirrhosis (Child-Pugh B and C) not listed for liver transplantation should be treated with the combination of SOF plus DCV and RBV for 12 weeks and for those patients presenting with contraindications to the use of RBV, EALS recommends extending SOF plus DCV treatment for 24 weeks (9). AASLD recommends SOF plus DCV with RBV for 12 weeks in patients with HCV genotype 3 infections who have decompensated cirrhosis, regardless of their status as candidates for liver transplantation, including those with hepatocellular carcinoma (10).

Four studies evaluated SOF plus DCV in patients with decompensated cirrhosis and HCV genotype 3 infection (19,23–25). The ALLY-1 study evaluated patients with compensated and decompensated cirrhosis or post-liver transplant recurrence treated for 12 weeks with SOF plus DCV and RBV. Among six patients with compensated/decompensated cirrhosis, five (83%) achieved SVR, which was slightly better in transplant recipients, 10 of 11 (91%) (24).

In an observational study, among 114 patients with decompensated cirrhosis treated with SOF plus DCV with RBV for 12 weeks, 70% achieved SVR. Seven patients treated with SOF plus DCV without RBV had a similar SVR rate, of 71% (25). In another observational study, cirrhotic patients with Child-Pugh B or C were divided in three treatment arms: SOF plus DCV for 12 weeks, SOF plus DCV for 24 weeks or SOF plus DCV with RBV for 24 weeks. The observed SVR rates were, respectively, 33% (2 of 6), 71% (12 of 17) and 70% (7 of 10) (19).

Finally, another observational study evaluated the SVR rates in patients with compensated or decompensated cirrhosis with HCV genotype 3 infection treated for 24 weeks with SOF plus DCV with or without RBV (22). Among those treated with SOF plus DCV, SVR rates were 100% for Child-Pugh A (19 of 19), 80% for Child-Pugh B (12 of 15), and 75% for Child-Pugh C (6 of 8). Conversely, among those who added RBV, response rates were 85% (11 of 13) for Child-Pugh A, 86% (12 of 14) for Child-Pugh B, and 100% (2 of 2) for Child-Pugh C (22).

## Conclusions

How can we best interpret the treatment responses for HCV genotype 3 treatments? To choose the best available therapeutic regimen is not simple, since some patient-related factors can negatively influence treatment outcomes, such as the presence of cirrhosis and previous treatment history for HCV.

**Figure 1:**
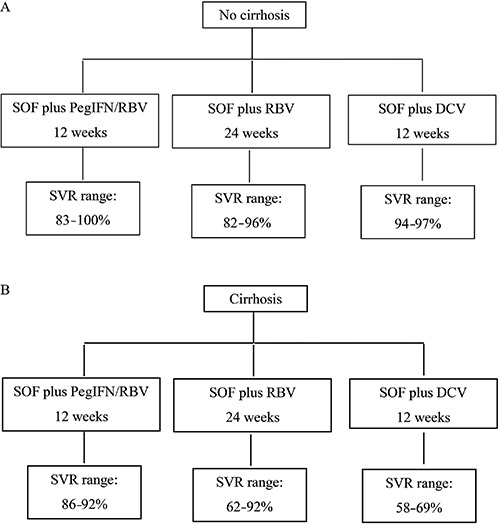
Sustained virological response (SVR) rates among patients with hepatitis C virus (HCV) genotype 3 infection. *A*, SVR rates among patients without cirrhosis and *B,* among patients with cirrhosis, according to different therapeutic regimens. PegIFN: pegylated interferon; SOF: sofosbuvir; DCV: daclatasvir; RBV: ribavirin.

The overall SVR rate obtained with SOF plus PegIFN/RBV for 12 weeks was high and no significant differences among cirrhotic and non-cirrhotic patients were observed (Figure 1). However, this therapeutic regimen includes PegIFN, a drug that requires subcutaneous injections, which can reduce adherence to treatment. Furthermore, the combination of PegIFN/RBV can cause several adverse effects that can lead to early treatment discontinuation, especially in cirrhotic patients, and it is contraindicated for a substantial number of patients (since previous studies have determined that approximately 17% of HCV-infected patients in the general US population had at least one contraindication to its use) (26,27).

The combination of SOF plus RBV for 24 weeks showed high SVR rates among naive patients with or without cirrhosis, but remains disappointing in treatment-experienced patients with cirrhosis. In addition, this therapeutic option has inconveniently longer treatment duration (24 weeks), which can increase costs and reduce patient adherence.

SOF plus DCV for 12 weeks provides very high SVR rates in non-cirrhotic patients (94–97%), but in those with cirrhosis the overall SVR of 59–69% is less than satisfactory. The addition of RBV increases the SVR rates in cirrhotic patients to above 80% and extending treatment to 24 weeks raises SVR rates to 90%. Nevertheless, it remains unclear if 12 weeks is enough for all patients or 24 weeks is the best option for some special populations, such as cirrhotic patients. For cirrhotic patients, the optimal duration, or even the best regimen, remains uncertain. Further studies are needed to clarify the best therapeutic regimen for patients with HCV genotype 3 infection.
